# A qualitative review of cannabis stigmas at the twilight of prohibition

**DOI:** 10.1186/s42238-020-00056-8

**Published:** 2020-12-07

**Authors:** Matt Reid

**Affiliations:** Department of Sociology and Criminology, Cabrini University, Radnor, USA

**Keywords:** Cannabis, Marijuana, Stigma, Stereotypes, Normalization, Privilege, Media, Review, Intersectionality, Sociology

## Abstract

**Background:**

As laws change and cannabis use increases, it is worthwhile to take a rich account of cannabis stigmas in society, and this review identifies a disjunction between quantitative investigations on cannabis users and qualitative investigations on the same population. This is also the first attempt to explicate cannabis stigmas as they manifest on multiple analytical levels. Following brief explanations of the normalization hypothesis and the concept of stigma, this review is organized between structural (macro) stigmas, social (meso) stigmas, and personal (micro) stigmas. Furthermore, since cannabis stigmas are similar to the stigmas faced by sexual minorities in that each is physically concealable, the two groups are compared here because the literature base is more extensive with the latter.

**Methods:**

This qualitative review synthesizes the body of empirical studies on both medical and nonmedical cannabis use with attention to stigma, stereotypes, and other social consequences. Studies considered for the review mostly come from the social sciences, particularly sociology. The information presented here is primarily drawn from peer-reviewed articles on cannabis users in the USA, though research from similar national contexts is cited as well.

**Results:**

This review suggests claims of normalization may be premature. While stigmas surrounding cannabis appear to have diminished, there is little evidence that such stigmas have entirely disappeared. It is possible that sweeping claims of cannabis normalization may be symptomatic of unchecked social privileges or social distance from cannabis users. Such claims may also be the product of valuing quantitative data over the nuanced accounts uncovered through qualitative investigations.

**Conclusion:**

This substantial coverage of the literature indicates the lived experience of a post-prohibition society is not the same as a one where cannabis is normalized. Individuals working with those who use cannabis should not assume stigmas have disappeared, especially since cannabis stigmas often intersect with other sources of social inequality. While a comprehensive discussion of ways to combat lingering social stigmas is beyond the scope of this review, it concludes by highlighting some of the strategies identified through research which help users resist or mitigate these oppressive forces. Future research would be wise to prioritize the experiences of people of color, women, and adult populations if the hope is to identify ways to further normalize the plant in American society.

## Background

As of the writing of this review, 11 states have legalized cannabis for adult use while 33 have made it available medically. Relatedly, the number of Americans using cannabis has been increasing year by year, with an estimated 15.9% using cannabis within the past year (SAMHSA 2019). Numbers like these suggest cannabis is becoming more commonplace in American society, but have things advanced far enough to say that cannabis is normalized? This qualitative review of the social scientific literature on cannabis suggests claims of normalization may be premature. While access and use rates are increasing, anti-cannabis stigmas are still formidable forces in the USA.

The purpose of this review has been to synthesize the body of empirical literature on both medical and nonmedical cannabis use with attention to stigma, stereotypes, and other social consequences. While medical patients and recreational users have different motivations for using cannabis and perhaps diverging experiences, their identity is similarly devalued since they are associated with the same plant. Furthermore, a prominent theme in the social science literature on cannabis is that of *blurred boundaries*, a term referring to the unclear line between medicine and recreational intoxicant (Ryan and Sharts-Hopko 2017; Satterlund et al. 2015; Bostwick 2012; Reinarman et al. 2011; Chapkis and Webb 2008; Osborne and Fogel 2008; Page and Verhoef 2006; Ogborne et al. 2000; Grinspoon 1999). There is a significant overlap between medical and recreational applications of cannabis, and people who use cannabis for medical purposes also situationally use their medicine in a manner similar to recreational users. The opposite is also true where all use can be seen as medical since the individual receives some health-promoting benefit, even if it is just relaxation. As such, these two subsets of cannabis users are combined in the ensuing review.

The information presented here is primarily drawn from empirical studies on cannabis users in the USA, though studies from similar national contexts are cited as well (Canada and the UK, in particular). These are occasionally supplemented with studies concerning the stigmas faced by sexual minorities in order to better illuminate the nature of cannabis stigmas. Each are concealable (or invisible) stigmas since they may not be immediately observable to onlookers, and comparisons to sexual minorities are somewhat common in the literature on cannabis users (Newhart and Dolphin 2019; Lau et al. 2015). The comparison is useful in explicating stigmas related to concealable traits, but it is nevertheless imperfect because sexual identity is best conceptualized as an ascribed status, something largely fixed within the individual. On the other hand, drug use—or more aptly, sobriety—is best conceptualized as an achieved status since it is something that can be altered through individual endeavors.

This is also the first known attempt to document cannabis stigmas as they manifest on multiple analytical levels. Following brief explanations of the normalization hypothesis and the concept of stigma, this review differentiates and explicates structural (macro) stigmas, social (meso) stigmas, and personal (micro) stigmas. Finally, while a comprehensive discussion of ways to combat lingering social stigmas is beyond the scope of this review, it concludes by highlighting some of the ways to resist or eliminate stigmas as identified in the social scientific literature.

## Normalization

The principle of normalization originally came from studies on disability (Wolfensberger and Tullman 1982) and has since been used as an interpretive tool for how cannabis is becoming an ordinary intoxicant in society (Sandberg 2012). The term is not used uniformly, though. Wolfensberger (2011) advocates that normalization should be understood as social role valorization for people who are at risk of social devaluation, where those with social stigmas are respected as filling valuable social roles. As applied to drugs, however, normalization is often taken to mean that a society’s attitudes and behaviors treat drug use as an unremarkable feature of everyday life (Pennay and Measham 2016; Parker 2005). While cannabis has become more commonplace in American society, this is only one of many components of the normalization process.

Largely starting in the 1990s, the normalization of cannabis has since become a prominent theme in sociological studies and is used to suggest that a society’s moral boundaries surrounding cannabis have been redrawn to accommodate non-abusive use (Duff et al. 2012; Sandberg 2012; Parker et al. 2002). The argument that “sensible” recreational drug use is normalized originated from longitudinal research on UK teenagers in the 1990s (Parker et al. 2002). Further developed by Parker (2005), normalization in the context of cannabis would take into consideration the following six dimensions: (i) the availability and accessibility of cannabis, (ii) cannabis trying rates and future intentions among nonusers to try cannabis, (iii) recent and regular cannabis use, (iv) the social accommodation of cannabis use where nonusers respect the right of others to use cannabis, (v) the cultural acceptance of cannabis where it is presented and understood as uncontroversial, and (vi) non-problematizing rhetoric and actions made by the government on cannabis (see also Pennay and Measham 2016; Duff et al. 2012).

Unfortunately, perhaps since some these dimensions are more easily quantified than others, claims that cannabis is normalized typically glance over the social and cultural dimensions. Indeed, critics have argued sweeping claims of normalization are oversimplified because social and cultural tolerance towards cannabis varies on a number of factors (Hathaway 2004). Since social contexts are largely ignored in statistical studies, normalization arguments need to do a better job of accounting for how different social groups perceive cannabis. For example, Erickson and Hathaway (2010) believe normalized cannabis use is a youth phenomenon. The concept of normalization originated from research on youth populations, and owing to how governments fund drug (ab)use studies, most empirical research on cannabis users focus on youth populations for the purposes of prevention (Garner 2016; Duff et al. 2012; Hammersley and Leon 2006). Yet perceptions about cannabis differ between generations so much so that age is one of the most powerful predictors of support for cannabis legalization (Elder and Greene 2019).

Another perspective in the normalization debate sees cannabis normalization as contextual since users navigate social settings of both normalized and stigmatized cannabis use (Lau et al. 2015). Others have acknowledged that the normalization process varies across social contexts (Hathaway et al. 2011), with Duff et al. stating: “Normalization ought to be understood as both broad social and cultural phenomena, as well as a disparate, contingent and uneven feature of local, micro-level, processes and contexts” (Duff et al. 2012:280). While no one denies that perceptions of cannabis vary between social settings and social groups, those who claim cannabis is normalized frequently generalize group-specific findings to the whole of society. More often than not, the groups on which such claims are based are those with social privileges (whites, males, and youths in particular) (e.g., Jarvinen and Demant 2011; Osborne and Fogel 2008; Parker et al. 2002).

It also might be wise to question the utility of applying the concept of normalization on a situational basis. Afterall, is not every deviant activity more or less normal depending on the social setting? Cannabis consumption is normal at a Cannabis Cup competition, just like public nudity is normal at a nude beach. This is why normalization is better understood as occurring on the societal and/or cultural levels. There has been some degree of cultural accommodation surrounding cannabis, but as this review will demonstrate, American society has yet to move beyond the stereotypes, stigmas, and structural penalties that have been endemic for so long.

## Stigma

In the social sciences, research on disgraced identities stems from the work of sociologist Erving Goffman and his influential book, *Stigma: Notes of the Management of Spoiled Identity* (Goffman 1963). Loosely defined, stigma describes a part of the self that is socially devalued to where it becomes morally offensive. This aspect can be a physical abnormality, faults of an individual’s character, or membership in a distasteful group. Either way, the “undesired differentness” (5) negatively distinguishes the individual from normal individuals in a society (Goffman 1963). This negative evaluation goes beyond individual sentiments and is a form of shared cultural knowledge, often making the stigmatized targets of socially acceptable prejudice and discrimination. As other scholars have built on Goffman’s foundational work, five defining features of stigma have so far been identified (Herek 2004). These include the following: (i) the endurance of stigmatizing features within individuals, (ii) the socially constructed meanings of the stigmatizing feature, (iii) the negative evaluation of the stigmatizing feature by society, (iv) the tendency of the feature to become a master status, engulfing the entire identity of a person, and (v) the oppression of stigmatized groups through the restriction of power, resources, and social rights.

Contemporary conceptualizations of stigma are becoming more attentive to socio-cultural contexts and variations in experiences among the stigmatized (Livingston and Boyd 2010). For example, instead of cannabis use carrying a uniform social stigma, some scholars argue the stigma surrounding cannabis now only applies to irresponsible use (Lau et al. 2015; Duff et al. 2012; Hathaway et al. 2011; Jarvinen and Demant 2011). In other words, moderate recreational use may have become normalized while excessive and/or dependent use is still problematized as drug abuse. As such, a “normal marijuana user” is one who practices self-control, discretion, and moderation (Duff et al. 2012). Similar sentiments have been found among medical cannabis patients who routinely emphasize the need for responsible use, often balancing symptom management with self-imposed limits on consumption during the workday (Newhart and Dolphin 2019). Hathaway et al. say, “A sense of normalcy is preserved by avoiding attributions and behaviors seen as risky, and thereby the associated stigma” (Hathaway et al. 2011:456). Any residual stigma may function to discourage abuse of marijuana, as “participants routinely insisted that this stigma had more to do with the circumstances of [marijuana] consumption than with the act itself” (Duff et al. 2012:281). This argument has its merits, but it conceals stigmatizing forces which oppress responsible and abusive users alike.

Once again, normalization theory applied to cannabis would assume there is no longer a powerful stigma associated with being a known cannabis user (Sandberg 2012; Wolfensberger 2011; Parker et al. 2002; Goffman 1963). While abusive use in certainly stigmatized, other studies suggest the stigma associated with the plant is applied to all users, no matter the context. For example, one striking finding from Satterlund et al. (2015) was that many medical cannabis patients used the word “stigma” unprompted by the interviewer. Their use of this word was consistent with how it is used in the social sciences, indicating the participants had an accurate understanding of the nature of stigma. They knew their medicine was viewed negatively by society and that their status as a user would result in judgment from others, despite their use of cannabis being medically justified.

While the stigma surrounding cannabis appears to have diminished, there is little evidence that such stigma has entirely disappeared. Medicalization and legalization certainly help reduce cannabis stigmas, but these transformations in policy do not entirely shift social perceptions on their own. As will be demonstrated in this review, the lived experience of a post-prohibition society is not the same as a one where cannabis is normalized. Such policies remove structural sources of stigmas, but since stigmas vary in their source, negative attitudes towards cannabis stem from a combination of overlapping institutional, social, and individual forces (Hammer 2015). The different sources and manifestations of cannabis stigmas are explored here. Since cannabis stigmas are similar to the stigmas faced by sexual minorities in that each is physically concealable, the two groups are occasionally compared here because the literature base is more extensive with the latter. Moreover, as stigmas interact on multiple analytical levels, research on stigmas applied to sexual minorities has differentiated between structural, social, and micro stigmas. The following is an attempt to do the same.

### Structural stigmas

Even though cannabis may be becoming more normalized, the fact that most users remain guarded about their use suggests there is a social structure or culture (or both) in which cannabis is stigmatized. *Structural stigmas* operate on the macro level and include cultural norms, state policies, and institutionalized procedures that oppress non-normal people (Livingston and Boyd 2010; Herek et al. 2009; Corrigan et al. 2006). Structural manifestations of stigma appear in institutional policies that restrict rights and diminish the life opportunities of people with stigmatized identities. Examples of this include state legislation that restricts firearm ownership and parental rights of people with mental illness (Corrigan et al. 2005), each of which are also applicable to cannabis users with the addition of being ineligible for organ transplants in most areas (Newhart and Dolphin 2019). Anti-sodomy laws are also an example of structural stigma as these were once used to justify the discriminatory treatment of homosexuals by organizations and individuals (Herek 2007). As is true with cannabis, “[c]riminalizing activities render them deviant, and it is generally assumed within society that there is a good reason for this status” (Bottorf et al. 2013:8).

Structural stigmas can also be ideological wherein cultural values function to oppress nonconformists. An example of this is *heterosexism*, a cultural default that presumes everyone to be heterosexual while problematizing visible sexual minorities as abnormal and inferior (Herek et al. 2009; Herek 2007). Similar sentiments can be found in society’s anti-drug hegemony. Hammer (2015) demonstrates that cultural norms impact policy decisions, and this can be seen when communities in states like Colorado and Michigan “opt-out” of cannabis businesses. Communities might oppose these businesses and the larger cannabis culture for a variety of reasons stemming from an ethos that prioritizes sobriety and productivity. Americans are said to have a uniquely Puritanical culture where pleasure achieved through intoxication is stigmatized and thus too are the tools to achieve these states (DeAngelo 2015; Earleywine 2010; Reinarman 1994). American capitalism as an extension of the protestant work ethic (see Weber 2011/1920) further stigmatizes intoxication because it is believed to hinder productivity, despite there being limited evidence that cannabis reduces performance (Hathaway et al. 2011; Earleywine 2010; Bonnie and Whitebread 1974). Aptly stated by author Michael Pollan:

Christianity and capitalism are both probably right to detest a plant like cannabis. Both faiths bid us to set our sights on the future; both reject the pleasures of the moment and the senses in favor of the expectation of a fulfillment yet to come—whether by earning salvation or by getting and spending. More even than most plant drugs, cannabis, by immersing us in the present and offering something like fulfillment here and now, short-circuits the metaphysics of desire on which Christianity and capitalism depend (Pollan 2001:175).

All in all, American society may stigmatize drugs because they conflict with the belief that pleasure should only be achieved through hard work, or by linking intoxication with immorality. This general drug stigma can crystallize into specific drug stigmas (Lloyd 2013), as is the case with “stoners” and “potheads.” The stigma of drugs is also one in which the individual is blamed for their irresponsible decisions and poor choices, framing drug-related stigmas as warranted or deserved (Satterlund et al. 2015; Lloyd 2013). Regardless of their exact manifestations, structural stigmas inform how individuals and groups view the stigmatized. Moreover, stigmas on this level serve to obscure facts by emphasizing immorality over logical reasoning. A consequence of this is that morally charged rhetoric often triumphs over objective, evidence-based arguments.

In sum, ideology and institutional policy work together to create structural prejudices and punishments that disadvantage stigmatized groups. As applied to cannabis, some structural sources of stigma include laws criminalizing cannabis, policies banning cannabis and cannabis users in the workplace as well as (public) housing, school programs where cannabis is taught to be a dangerous drug, and organizational views that problematize cannabis, such as those found within Child Protective Services and many health care services. More importantly, these macro-level forces create the context for stigmatizing actions and beliefs within groups and individuals.

### Social stigmas

*Social stigmas* work on the meso level and describe how organizations and groups endorse cultural messages that disadvantage stigmatized people (Livingston and Boyd 2010). Yet there is some disagreement between scholars on the exact terminology used at this analytical level. Corrigan et al. (2006) call this *public stigma* whereas Herek et al. (2009) call this *enacted stigma*. Regardless, this type of stigma is overt and manifests in both group and individual actions including epithets, shunning, ostracism, discrimination, and violence towards the stigmatized group (Herek 2007). A good example of a social stigma related to cannabis is the belief that it is incompatible with social role expectations—that those who use cannabis in the roles of parent, student, and/or worker are seen as less proficient in these roles as their non-using peers (Hathaway et al. 2011). Parents who use cannabis may be shunned by other parents, students who use cannabis may be forced to complete a rehabilitation program, and workers who use cannabis may be fired. Even if no action is taken, the sentiment often results in heightened scrutiny towards cannabis users where any minor mistake is directly attributed to cannabis intoxication (Newhart and Dolphin 2019).

Social stigmas also arise when a specific group’s norms are violated, causing the group to react negatively towards the norm violation. This phenomenon can be observed when those in the American medical establishment scorn the idea of cannabis as a medicine. Overall, the authoritarian nature of mainstream medicine stigmatizes cannabis—perhaps viewing it as competition—because it was remedicalized through a grassroots, patient-led movement (Penn 2014; Kondrad and Reid 2013; Chapkis and Webb 2008; Jones and Hathaway 2008). The establishment also takes issue with how cannabis as a medicine has not undergone the rigorous clinical trials required for every other medicine, despite there being a plethora of evidence from other methods which support cannabis’ efficacy for a range of ailments. Moreover, the FDA’s standard regulatory approval process favors isolated chemical compounds, making it unsuitable for a multi-cannabinoid plant like cannabis (Frye 2018; Webb and Webb 2014; Bostwick 2012; Grinspoon 1999). In this way, cannabis as a medicine side steps the edict of formalism within mainstream American medicine, inviting condemnation from medical professionals.

One of the most powerful purveyors of cannabis social stigmas is the media (Mortensen et al. 2019). Leading up to the federal prohibition of marijuana in 1937, Harry Anslinger and the Federal Bureau of Narcotics influenced the publication of numerous propaganda articles whereas anti-marijuana articles before this time were largely nonexistent (Griffin et al. 2013; Becker 1973/1963; Bonnie and Whitebread 1974)^1^. These articles helped garner public support for prohibition as they sensationally linked marijuana to crime, violence, sexuality, and immorality. Unfortunately, this sentiment survives today though perhaps in a much subtler context. While research has found a trend in positive marijuana reporting starting in the early 1990s (Stringer and Maggard 2016), the common inclusion of law enforcement personnel as experts in marijuana articles still imparts the feeling that something is criminogenic about the substance (Mortensen et al. 2019; Kim and Kim 2018; Haines-Saah et al. 2014; Boyd and Carter 2012)^2^. Seemingly playful stoner stereotypes are also detrimental to the public’s perception of cannabis since they make users appear incompetent and immature:

[B]ecause cannabis use is also associated with the young adult phase of the life course, stereotypes of cannabis users exaggerate qualities associated with young adults, such as lock of experience and irresponsibility, while also implying that cannabis users who have aged out of this phase of the life course are immature or “burnouts” because they have maintained inappropriate behaviors relative to their age (Newhart and Dolphin 2019:189).

Social stigmas can also be directed towards those who associate with the stigmatized group, resulting in what Goffman (1963) called *courtesy stigma*. This occurs when an individual is stigmatized for who they associate with rather than their own group affiliations or behaviors. For example, Brainer (2015) found straight teenagers with LGB (lesbian, gay, bisexual) siblings where frequently accused of being homosexual by their peers. Even though the teens interviewed were straight, their heterosexuality was called into question by their peers for having an openly LGB sibling. Using similar reasoning, a courtesy stigma may also be applied to non-cannabis users who have friends or family whose cannabis use is publicly known. The group association is what is targeted rather than something about the individual or their behaviors. For instance, parents may diligently conceal their use of cannabis from their child’s friends (or even their own children) so that their child does not get the reputation of having stoner parents (Newhart and Dolphin 2019). More evidence of courtesy stigmas comes from organizational research which found cannabis stigmas are often transferred to businesses who support the regulated cannabis industry, such as law offices, accounting firms, and public relations agencies (Lashley and Pollock 2019). Relatedly, research on medical cannabis cultivators discovered many workers use “cover stories” when conversing with strangers or community outsiders. These individuals often say they work in a similar field or generically express they are small business owners (Adelman 2013).

Bottorf et al. (2013) identified three other reasons that social stigmas are directed towards medical cannabis patients, in particular. The first of these was negative views of cannabis as a recreational drug, where larger society constructed patients as “potheads” or “stoners” (Bottorf et al. 2013). Patients reported friends and family members explicitly doubted the severity of a patient’s illness and motives for cannabis use. Yet patients can avoid some of this stigma by clarifying how they use cannabis as a prescribed medicine rather than as a tool for pleasure (Ko et al. 2016; Bottorf et al. 2013). The way a patient medicates can also impact this stigma as medical cannabis is more stigmatized when the method of administration mirrors recreational use styles—joints and water pipes, for example (Rudski 2014). Nonetheless, this stigma forced patients to conceal their cannabis use from their social networks, withdraw from disapproving family and friends, and sometimes even relocate to another area (Satterlund et al. 2015; Bottorf et al. 2013).

The second source of medical cannabis stigmas was a presumption that patients were engaged in illicit drug activity (Bottorf et al. 2013). Patients who cultivated their own medicine were suspected of being drug dealers and reported repeated harassment by police, landlords, and housing authorities for those in state-subsidized housing. Women also have the added stigma of being labeled negligent mothers if it was known they consume cannabis, a well-founded fear in light of child protective services intervening in the homes of families who use medical cannabis (Reinarman et al. 2011; Boyd 2009).

The third and final source of stigma confronting cannabis patients was using cannabis in the context of layered vulnerabilities (Bottorf et al. 2013), or how cannabis use can be entangled in a web of other marginalized identities. For example, cannabis patients sometimes suffer from controversial illnesses that carry their own stigmas (Newhart and Dolphin 2019; Scrambler 2009), such as fibromyalgia, HIV/AIDS, or mental illness. Likewise, those in poverty, as well as gender and sexual minorities, may already be framed as “problem patients” by the larger medical establishment, and their use of cannabis further adds to their social devaluation (Bottorf et al. 2013; Chapkis and Webb 2008; Werner 2001). Indeed, there is a tendency for medical professionals to spend time analyzing the patient’s moral fiber and addictive potential rather than the patient’s underlying illness (Dubin et al. 2017). This takes time away from meaningful care and discourages patients from being honest with their care providers. Dubin et al. (2017) suspect there is a mismatch between physicians’ formal curriculum, which emphasizes stigma reduction, and the hidden curriculum, which implicitly reproduces anti-cannabis attitudes. Furthermore, when patients forgo medical cannabis over concerns of stigma, they may experience unnecessary pain, suffering, and unwarranted stress (Victorson et al. 2019; Ryan and Sharts-Hopko 2017). “Medicine can only be effective if it is taken, and stigma and lack of acceptability can interfere with compliance and safe access” (Rudski 2014:318).

This convergence of stigmatizing forces within the same individual is known as *intersectional stigma* (Turan et al. 2019). Intersectionality acknowledges people do not experience power relations based on one identity at a time. Rather, humans experience a simultaneous plurality of social privileges and oppressions that intersect in unique ways (Collins and Bilge 2016). Medical cannabis patients face devaluation owning to their use of cannabis and their underlying health condition, but intersecting inequalities are present in nonmedical users as well, and this fact alone should trouble sweeping claims that cannabis is normalized.

Layered vulnerabilities are particularly sensitive in the USA with its history of socially constructed drug scares. Recurring moral panics related to drugs are often accompanied by ideologically constructing minority groups as responsible for drug-related social problems (Reinarman 1994). In terms of cannabis, this has been done with Mexican immigrants, jazz musicians, countercultural youth, and AIDS patients who campaigned to remedicalized in the 1990s. All of these groups, at one or more points in American history, served as a “dangerous class” upon which the government justified continued cannabis prohibition. This is why sociologist Craig Reinarman calls drugs “richly functional scapegoats” (Reinarman 1994:165) for social fears rooted in racism, xenophobia, classism, ageism, and more.

A consequence of this is the stigma of being a cannabis user is differentially applied and experienced between social classes, races, genders, and generations (Hammer 2015; Satterlund et al. 2015; Bottorf et al. 2013; Reinarman et al. 2011; Hathaway 2004). Those with social privileges are often able to better conceal their stigmas or resist stigmatizing labels becoming their master status. Indeed, cannabis may be more normalized for those with elite statuses including musicians, athletes, and other celebrities. These individuals were relatively immune from the “othering” endemic in Canadian newspaper articles on cannabis use where Haines-Saah et al. (2014) uncovered a “tendency to use a person’s race, class, or other irrelevant characteristics to position him or her as different, problematic, or ‘other’” (55). Likewise, cannabis use by women and racial minorities is more stigmatized relative to men and whites. Research has demonstrated women take extra precautions to remain discreet when using they typically face harsher criticism than men: “[B]ecause marijuana is often seen as a masculine activity, a normalized marijuana using image is unavailable to many women” (Mostaghim 2019:64).

While some studies suggest being a cannabis user no longer denotes a master status and has instead become just another aspect of a person’s life (Jarvinen and Ravn 2014; Sandberg 2012; Hathaway et al. 2011), this has not exactly been the case for people of color. Sports reporters commonly dismiss or trivialize cannabis use by white athletes while intensely scrutinizing cannabis use by black athletes (Dickerson 2018; Lewis and Proffitt 2012). Many black athletes whose cannabis use was leaked by the media are subsequently described as thugs or lacking discipline, even in non-cannabis-related stories about their behavior. A recent content analysis of Colorado news reporting adds evidence to this racist phenomenon, finding that legalization did not result in a normalized depiction of minority cannabis users (Mortensen et al. 2019). When criminal or stoner stereotypes were used by the media, they were more likely to feature racial minorities than white people^3^.

Considering how cannabis stigmas intersect with social inequalities troubles sweeping claims that cannabis is normalized. It is even possible such claims come from a colorblind position of racial privilege. For a whole host of reasons from racism to implicit biases, society intensely vilifies drug use when the user lacks white skin (Tiger 2017; Alexander 2010). This double standard has repercussions beyond political correctness in the media. American legal scholars Bender (2016) and Vitiello (2019) argue that campaigns to legalize cannabis have so far failed to focus on racial equity despite the enforcement of prohibition being largely racialized. With the exception of Washington D.C., racial justice was rarely mentioned as a reason why cannabis should be legal for medical or adult use. In fact, when race was brought into the pro-legalization debate, it was often in the form of disdain for Latin American drug cartels. With this racialized menace in mind, “voters may have been making the choice between their perception of shady cartels of color controlling the illicit market, and of more trusted white business owners and local government profiting from marijuana consumption” (Bender 2016:694). The best way to mitigate the racist legacy of prohibition may be to explicitly confront them with non-stigmatizing, color-conscious advocacy.

### Micro stigmas

At the individual level, stigma can be felt, internalized, or both. *Felt stigma* results from the awareness that an identity is culturally devalued and can result in anticipatory behavior to avoid negative interactions (Herek et al. 2009; Herek 2007). Knowing that something carries a stigma causes behavioral adjustments in both the stigmatized and normative groups. In the context of heterosexism, both heterosexual and non-heterosexuals may avoid gender nonconforming behavior and deviant sexual expressions in order to avoid the label of a stigmatized sexual identity (Herek et al. 2009; Pascoe 2007). Among cannabis users, concealment strategies to evade stigma include substituting smoking with edibles, using eye drops, lighting incense, changing clothes after smoking, consuming mints, and keeping vigilant about who else could be watching (Giombi et al. 2018; Lau et al. 2015; Bottorf et al. 2013). Additionally, some users refrain from commenting on cannabis-related issues when they arise in everyday conversations (Satterlund et al. 2015).

All of this is done to avoid suspicions that one is a cannabis user. While not unreasonable, these precautionary measures can be stressful for the self and one’s intimate relationships. Similar to Karp’s (2006) findings on how felt stigma effected on the romantic lives of individuals taking antidepressants, individuals who use cannabis worry how it might impact potential relationships (Lau et al. 2015). While some are forthcoming to new partners, both medical and nonmedical users experience stress when considering how to disclose their use of cannabis.

Felt stigma may lead to enacted stigma when an individual is motivated to publicly prove they are not part of a stigmatized group. For example, Pascoe (2007) found that teenage boys routinely rebuke homosexuality in order to reaffirm their heterosexuality to others. Using “fag” as an insult (an example of enacted stigma) signals to others that one is not a fag, even if the target of the abuse has nothing to do with homosexuality (Pascoe 2007). Anticipating rejection from others, both cannabis users and non-users may avoid or ridicule cannabis culture, individuals known to consume cannabis, and anything else that may raise suspicions of cannabis use (Lau et al. 2015; Satterlund et al. 2015).

Research by Corrigan et al. (2006) proposes a similar model using the language of stereotypes instead of stigma. They call social stigma *stereotype awareness* in that people know negative attitudes towards some identities exist within society. After becoming aware of these negative evaluations, an individual may start to endorse such beliefs through *stereotype agreement*. If this occurs and the individual applies devalued cultural stereotypes onto themselves, it may decrease one’s self-esteem and self-efficacy (Corrigan et al. 2006). This is also known as *internalized stigma* and results when individuals come to believe in the negative cultural messaging surrounding a stigmatized identity (Livingston and Boyd 2010).

Both the stigmatized and normative groups may internalize cultural stigmas, resulting in *self-stigma* in the former and *prejudice* in the later (Herek et al. 2009; Herek 2007). Heterosexism can be endorsed by heterosexuals and manifests as prejudice towards non-heterosexual people. The same heterosexist beliefs can also be accepted by non-heterosexuals and manifest as internalized homophobia, internalized heterosexism, and internalized homonegativity (Herek 2007). As with cannabis, users may tacitly accept mainstream cultural assumptions that problematize cannabis and users (Hathaway et al. 2011). All of these result in self-directed prejudice on the part of stigmatized individuals as they internalize society’s negative evaluation of their identity (Herek et al. 2009). However, Corrigan et al. (2006) note that not all people who are aware of or endorse cultural stigmas will internalize such stigmas, and this resistance protects their self-esteem and self-efficacy. People have an agentic capacity for cognitive resistance and may fight against the moral judgments of others, and this has been documented among medical cannabis users (Newhart and Dolphin 2019). However, felt stigma does not necessarily have to be internalized as it results from the mere knowledge that an identity carries negative social consequences.

There is a dearth of research on stereotype agreement and internalized stigma among cannabis users. However, research exists on cannabis-related *stereotype threats* which occur when individuals believe they may be stereotyped by others, creating a pressure that causes the individual to adjust their appearance and/or behavior (Newhart and Dolphin 2019; Hirst et al. 2018; Looby and Earleywine 2010). For example, psychological research on stereotypes has found males are more likely to be judged as cannabis users than females (Hirst et al. 2018). Indeed, most American cultural images of stoners are males, perhaps making men more vulnerable to cognitive distress over their cannabis use (Looby and Earleywine 2010). Stoner stereotypes are also largely associated with youth and young adults, linking adult use of cannabis to immaturity and other age-inappropriate behaviors (Mortensen et al. 2019; Newhart and Dolphin 2019). Indeed, the stoner stereotype exaggerates qualities like irresponsibility, laziness, and lack of experience. This is good to keep in mind because many academic studies on cannabis use are based on youth samples. Resulting data may help reinforce cultural assumptions related to age and cannabis use, essentially confirming rather than challenging the stereotype.

The internalization of stigma is distinct from an isolated individual attitude since stigma constitutes shared cultural knowledge. Furthermore, how an individual acquires a stigmatizing label is not uniform and depends on the specifics of the situation. For example, Herek (2004) accurately points out that homosexual behavior may be dismissed if it only occurs in adolescence, under the influence of alcohol or drugs, or within the confines of a sex-segregated institution like prisons. Anecdotally, the same may also be true with cannabis. Intermittent cannabis use during one’s teenage years may be forgiven if the individual ceases use at some point before adulthood. The stoner label might also be avoided by saying one only smoked when they were drunk, by claiming they mistook a joint for a cigarette, or that they unknowingly once ate food infused with cannabis. Society may also not apply the stoner label to individuals who admitted to using cannabis only while on vacation in Jamaica, Amsterdam, or another location with a well-known cannabis culture. In all these cases, cannabis use is seen as incidental and not characteristic of the individual (see Herek 2004 for a discussion on how stigmatizing labels may be withheld).

Making matters more complicated, stigmas vary in their degree of public visibility. Goffman (1963) termed visible stigmas *discrediting* and non-visible stigmas as *discredible*. Since the latter is concealable and is the case with cannabis users, an individual is only devalued once their stigma becomes known to others. People with non-visible stigmas routinely manage the extent to which others are aware of their stigma (Herek 2004; Goffman 1963). This is a strategy that protects the self on the one hand but may also diminish self-esteem and self-efficacy on the other. For example, one study found negative self-perceptions were significantly lower among LGB individuals who were out to family and friends compared to LGB individuals who merely believed their family and friends knew their closeted sexual identity. This reduction of self-stigma is highest for those who had explicit conversations about their sexual identity (Herek et al. 2009). As such, while keeping a stigmatized identity a secret may protect against negative reactions from others, it may also damage one’s sense of self and mental health. After all, our sense of self is partly formed by those who we find emotionally important. We internalize the expectations and opinions of others, creating “a chorus of voices that shape our internal conversations about who we are, what we ought to feel, and how we should act” (Karp 2006:126).

The discussion above hints at the difference between *normification*, which describes how the stigmatized assimilate into society by attempting to pass as normal, and *normalization*, which describes the societal transformation wherein a once-stigmatized identity becomes acceptable or even celebrated (Wolfensberger 2011; Goffman 1963). For example, normifying techniques are evident in a study of cannabis tourism in Colorado where the industry consciously tries to challenge perceptions of deviance through maintaining a professional appearance and conveying the latest scientific research on cannabis to their guests (Keul and Eisenhauer 2019). Professionalism is generally a good business practice and undoubtedly facilitates cannabis normalization. Nonetheless, some scholars have suggested that the normifying strategy of concealment also normalizes cannabis (Lau et al. 2015; Johnson et al. 2008). This is somewhat reminiscent of the closet in the LGBTQ community, something that both oppresses self-expression and diminishes the group’s visible presence in society. Concealment may be vital when protecting the self from structural penalties or when respecting the personal space of non-users, but if such tactics are undertaken in fear of rebuke from intolerant others, concealment may ultimately impede the transformation of social expectations necessary for normalization.

## Resistance and empowerment

The extensive evidence of cannabis stigmas troubles sweeping claims that cannabis is normalized. While sentiments towards cannabis have been improving in American society, it is doubtful that cannabis use is no longer a source of status loss. This is why resisting stigmas is important, as stigmatized people often face a vicious cycle of compounding disadvantages. Stigmas are associated with lower levels of hope, self-efficacy, self-esteem, social support, and quality of life (Livingston and Boyd 2010). Stigmatized people may respond with anger, self-imposed isolation, or attempts to conceal their identity, all of which are associated with negative mental health outcomes (Ahern et al. 2006). So how can stigmatizing forces be resisted or combated?

From a psychological standpoint, self-stigma can be reduced through one of two general strategies (Mittal et al. 2012). The first strategy is to alter stigmatizing beliefs and attitudes in an individual through practices such as cognitive restructuring and psychoeducation. The second strategy, which is gaining popularity among stigma experts, is one of mindful acceptance. This latter approach does not challenge stigmatizing forces or stereotypical thinking, but instead aims to make the individual more comfortable with their current self. Mindful acceptance can enhance a person’s overall self-esteem, empowerment, and help-seeking behavior (Mittal et al. 2012). Basically stated, others will judge a person for using cannabis, but since there is little that person can do to change the perceptions of others, it is best for that person to not waste their time worrying about it.

Yet the most effective stigma reduction strategy may be in changing societal beliefs and attitudes (Herek et al. 2009). If stigma is conceptualized as being a top-down model wherein individuals internalize negative cultural stereotypes, it makes sense to enact destigmatizing changes on the structural, cultural, and organizational levels. Changes in drug policy may help shift cultural frameworks surrounding cannabis by stopping institutional messages aimed at presenting users as morally deficient, criminal, or psychologically ill (Hathaway et al. 2011). Such structural changes can be accomplished through legal reform, but also through educating institutional leaders and publicly addressing the consequences of cannabis’ criminalization (Bottorf et al. 2013). This last element—tackling the legacy of prohibition—is particularly important as legalization by itself may not meaningfully reduce stigmas for those with less social privileges. A good example of this can be found in a statement by Anqunette Sarfoh, a former news anchor who quit her job to open a medical cannabis dispensary. Here she reflects on the challenges in recruiting diverse staff in the city of Detroit:

In our community, cannabis use has been stigmatized, because of how the legal impacts have affected our community… In [white] communities, kids can go in a cornfield and smoke a joint and go on about their lives. But in our communities, what happens when you're caught, your future is gone. And so for the longest time, you just don’t even touch it and you grow up knowing that it could ruin your life (Anqunette Sarfoh quoted in Gray 2019).

Even after cannabis is legalized, the trauma resulting from prohibition will continue to exist as will entrenched anti-cannabis ideologies. Thankfully, many cannabis users already exhibit *tertiary deviance* (Bottorf et al. 2013), defined as deviants who fight to change social stigmas related to their identity. Primary deviance is characterized by denial, secondary deviance is categorized as acceptance, but tertiary deviance is characterized by advocacy and mobilization (Adler and Adler 2016/1994; Becker 1973/1963). Empowered individuals may become public representatives for their stigma (Lloyd 2013; Goffman 1963), a situation now evident with celebrities sharing their stories of using medical cannabis or cannabis for pleasure. Nonetheless, there seems to be a greater need for public representations which reflect the diversity within the cannabis community rather than the familiar white men in suits.

Similarly, Newhart and Dolphin (2019) propose three ways cannabis stigmas can be eroded. The first is the public identification of cannabis users so that their diversity becomes visible to others. A visible cannabis community would challenge flattening stoner stereotypes which assume all users have similar negative qualities. Indeed, cannabis users are a heterogeneous group with a significant degree of diversity (Loflin and Earleywine 2014; Osborne and Fogel 2008). Society may discover, for example, that high achievers like graduate students and college professors use cannabis regularly (Garner 2016). This public identification will also reveal how cannabis users can be found within everyone’s social network, which is the second strategy proposed by Newhart and Dolphin (2019). Non-users may learn that they have respected friends, family, and associates who use cannabis. This can be seen in the study by Bottorff et al. where one participant transformed her once-disapproving mother into a “full on cannabis granny” (Bottorf et al. 2013:7).

Finally, stereotypes can be challenged by identifying the source or perpetrator. Pointing out how the government, media, and others create and reinforce these misconceptions dispels the stereotype’s naturalness. In other words, these devaluations and characterizations are social constructions, not hard truths supported by objective evidence. In fact, participants in several studies thought much of the stigma surrounding cannabis use is a result of societal ignorance or deliberate misinformation (Satterlund et al. 2015; Hathaway et al. 2011). Nonetheless, while challenging deceptive sources of information about cannabis is a good practice, the general public’s perception of drug use is also shaped by their personal experiences and their surrounding environments. Spaces that facilitate contact between the public and the stigmatized can change negative attitudes, relying on the principle that with familiarity comes empathy (Lloyd 2013; Herek 2007). As such, creating regulated spaces for social use may not only be of service to individual cannabis users, but these spaces may also help normalize cannabis within larger society.

## Conclusion

In his exploration of the deep history of cannabis throughout the world, John Charles Chasteen concludes it has been a substance associated with outsiders in every society. Until the latter part of the twentieth century, cannabis was “used by the poor, by the marginal, by the chronically ill, by the artistically and philosophically and spiritually inclined, by seekers after the meaning of life, and by social and religious nonconformists of various stripes” (Chasteen 2016:137). Stated otherwise, the deviant status of cannabis may be universal as groups associated with the plant are traditionally positioned apart from respectable society. The degree of this deviance varies over time and space, but considering this historical constant, scholars should be careful of claiming cannabis normalized unless the body of evidence thoroughly supports such a claim. As has been demonstrated in this qualitative review of cannabis stigmas, the deviant status of cannabis in American society still appears to hold true. Even today with cannabis, the “worst consequences, social and individual, seem to arise from how nonusers react to users” (Becker 1973/1963:200).

The debate surrounding cannabis normalization is ongoing and much work still needs to be done in the area. Though this review focused on cannabis users as a single class, it may be interesting to examine if stigmas differ between occasional and frequent users. Furthermore, most of the literature on cannabis normalization focuses on the macro, rather than micro, levels of analysis (Duff et al. 2012). There is little research on how social groups or individuals come to change their views on cannabis, though there is some evidence that institutional change requires activism and patience. Normalizing a stigma necessitates a significant cultural shift often occurring over many years or decades. As such, the intent here was not to rain on the parade of cannabis activists who have steadfastly progressed the status of the plant. Instead, the intention has been to show that once a stigma is established within a society, it is very difficult to change or remove. The still-marginalized status of sexual minorities once again demonstrates this phenomenon.

In sum, prevailing cannabis stigmas problematize linear narratives of normalization. No one doubts remarkable improvements have been made in recent times, but claims that cannabis is normalized at the societal level may be premature. Such claims may be symptomatic of unchecked social privileges or social distance from cannabis users. They may also be the product of valuing quantitative data over the nuanced accounts gained through qualitative investigations. Rates of legalization and use statistics do not capture the often-subtle struggles those in the cannabis community face in their everyday lives. As such, those investigating this topic should be careful not to overgeneralize arguments about normalization unless they can account for quantitative and qualitative changes among diverse groups and social settings. Significantly more attention should be placed on the dimensions of cultural and social accommodations in the normalization hypothesis. Furthermore, future research on cannabis stigmas should strive to incorporate and empower the most vulnerable in the community. Doing this will not only protect against making privileged claims to normalization, but it may also serve to identify meaningful strategies to further normalize the plant in society.

## Data Availability

Not applicable.

## Footnotes

1 For example, from 1851 to 1930, only 8 articles with “marijuana” or “marihuana” appeared in the *New York Times*. In the next decade alone (1931-1940), this number jumped to 133 articles (Griffin et al. 2013). Becker (1973/1963) uncovered a similar pattern using the *Reader’s Guide to Periodical Literature* which indexed popular magazines. From July 1937 to June 1939, 17 magazine articles were published on marihuana, 10 of which were produced by the Federal Bureau of Narcotics.

2 Analyzing 640 articles published in 10 newspapers between 1995 and 2014, Kim and Kim (2018) revealed 44.5% contained the perspective of law enforcement.

3 Analyzing 458 images from Colorado newspapers, Mortensen et al. (2019) and found racial minorities were depicted as criminals more than whites (23.5% to 13.4%), and that racial minorities were depicted as displaying more pot-culture stereotypes than whites (34.6% to 16.5%).
